# Association of Ki-67 Labelling Index and IL-17A with Pituitary Adenoma

**DOI:** 10.1155/2018/7490585

**Published:** 2018-05-31

**Authors:** Brigita Glebauskiene, Rasa Liutkeviciene, Alvita Vilkeviciute, Inga Gudinaviciene, Aurelija Rocyte, Dovile Simonaviciute, Ruta Mazetyte, Loresa Kriauciuniene, Dalia Zaliuniene

**Affiliations:** ^1^Lithuanian University of Health Sciences, Medical Academy, Department of Ophthalmology, Lithuania; ^2^Neuroscience Institute, Lithuanian University of Health Sciences, Medical Academy, Lithuania; ^3^Lithuanian University of Health Sciences, Medical Academy, Department of Pathology, Lithuania; ^4^Lithuanian University of Health Sciences, Medical Academy, Department of Laboratory Medicine, Lithuania; ^5^Lithuanian University of Health Sciences, Medical Academy, Lithuania

## Abstract

The aim of the present study was to determine if the Ki-67 labelling index reflects invasiveness of pituitary adenoma and to evaluate IL-17A concentration in blood serum of pituitary adenoma patients. The study was conducted in the Hospital of Lithuanian University of Health Sciences. All pituitary adenomas were analysed based on magnetic resonance imaging findings. The suprasellar extension and sphenoid sinus invasion by pituitary adenoma were classified according to Hardy classification modified by Wilson. Knosp classification system was used to quantify the invasion of the cavernous sinus. The Ki-67 labelling index was obtained by immunohistochemical analysis with the monoclonal antibody, and serum levels of IL-17A were determined by enzyme-linked immunosorbent assay (ELISA). Sixty-nine PA tissue samples were investigated. Serum levels of IL–17A were determined in 60 patients with PA and 64 control subjects. Analysis revealed statistically significantly higher Ki-67 labelling index in invasive compared to noninvasive pituitary adenomas. Median serum IL-17A level was higher in the pituitary adenoma patients than in the control group.* Conclusion*. IL-17A might be a significant marker for patients with pituitary adenoma and Ki-67 labelling index in case of invasive pituitary adenomas.

## 1. Introduction

**Figure 1:**
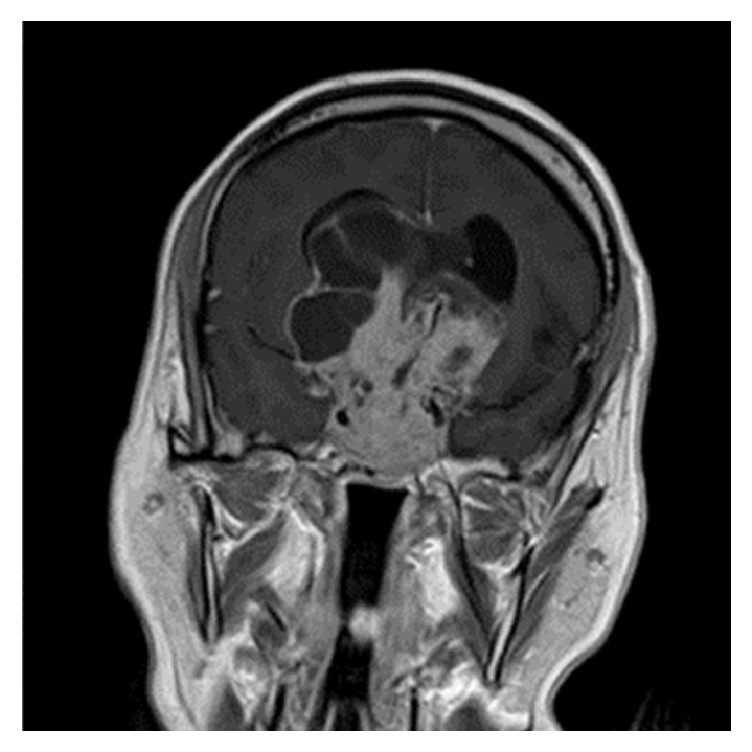
Invasive pituitary adenoma.

Pituitary adenoma (PA) is an intracranial tumour arising from the hormone-secreting epithelial cells in the anterior lobe of hypophysis with reported estimated prevalence rates ranging between 14.4% and 22.5% in pooled autopsy and radiological series, respectively [1]. PA is covered by dura and is located in the hypophyseal fossa of the sphenoid bone and surrounded by the two cavernous sinuses laterally [2]. Although PA is considered as a benign tumour, it frequently invades the sphenoid bone and the cavernous sinus [3–10], and if the direction of expansion is suprasellar, it compresses the optic chiasm [11–19]. About 35% of all pituitary neoplasms are considered as invasive tumours (Figure 1) [9]. PA can transform to a malignant tumour as well [20]. However, PAs can easily expand laterally without invasion, because the medial wall of the cavernous sinus is a thin dural bag [21, 22]. It is important to differentiate the invasive PA growth from lateral growth without invasion because it can influence treatment and prognosis of PA [23].

In order to prognosticate the course of PA, attention has been drawn to the scientific surveys about the invasiveness markers, such as Ki-67 labelling index (LI), IL-17A, and several others [24–26]. The Ki-67 is supposed to be one of the invasiveness markers of PA [5, 10, 24, 26–32]; despite this fact, some studies have shown controversial data [33–35]. It has been found that IL-17A plays an important role in the development of various tumours, for example, breast cancer, hepatocellular carcinoma, and cervical cancer [36–39]. However, some studies have revealed that IL-17A inhibits the development of tumours [40, 41] and there is only one survey that analyses the IL-17A impact on the PA growth [42, 43]. We also hypothesized that the development of PA can be influenced by the concentration of IL-17 A.

Therefore, the aim of the present study was to determine possible molecular markers which may serve as diagnostic and prognostic PA markers.

## 2. Materials and Methods

Permission to undertake the present study (number P2-9/2003) was obtained from the Biomedical Research Ethics Committee of Lithuanian University of Health Sciences (Kaunas, Lithuania). The study was conducted in the Departments of Ophthalmology, Neurosurgery, and Laboratory Medicine, Lithuanian University of Health Sciences Hospital (Kaunas, Lithuania).

Sixty-nine PA tissue samples were investigated. Serum levels of IL–17A were determined in 60 patients with PA and 64 control subjects. Specimens of PA and blood serum were taken before treatment.

The inclusion criteria were as follows: (1) determined and confirmed PA via MRI; (2) patient's general good condition; (3) patient's consent to take part in the study; (4) age** ≥** 18 years; (5) no other brain tumours or tumours with other localizations, intracranial infection, demyelinating lesions, or cerebrovascular disease.

The control group for IL-17A evaluation was made of 64 healthy subjects, who were admitted to the Hospital of Lithuanian University of Health Sciences Department of Ophthalmology for preventive ophthalmological evaluation, considering the patient's age and gender in the PA group. Therefore, the medians of patient age in the control group and in the PA group did not differ statistically significantly (p<0.05). Demographic and clinical data are shown in Table 1.

*Brain Imaging*. All pituitary adenomas were analysed based on magnetic resonance imaging (MRI) findings. The preoperative MRI investigations were performed with 1,5 T MRI scanners (Siemens MAGNETOM Avanto, 1,5 T Philips ACHIEVA) using a head coil and a standard pituitary scanning protocol, obtaining T1W sagittal and coronal and T2W/TSE coronal precontrast images, and T1W coronal and sagittal Gadolinium-enhanced MR images with the intravenous agent gadodiamide (Omniscan, GE Healthcare). The retrospective analysis of MRI data was conducted by an experienced radiologist. The suprasellar extension and sphenoid sinus invasion by PA were classified according to the Hardy classification, modified by Wilson (Table 2) [23]. The degree of suprasellar and parasellar extensions was graded as stages A–E. The degree of sellar floor erosion was graded as grades I-IV. Grade III shows localized sellar perforation, and grade IV shows diffuse destruction of sellar floor, which are the signs of invasive PA. The Knosp classification system (Table 3) [5] was used to quantify the invasion of the cavernous sinus. Grade 3 and 4 pituitary tumours were considered to be invasive.

*Ki-67 labelling index*. The Ki-67 LI was obtained by performing an immunohistochemical analysis with the monoclonal antibody (clone SP6;* Spring Bioscience Corporation*). The Ki-67 LI was defined as the percentage of positive staining tumour cells.

**Figure 2:**
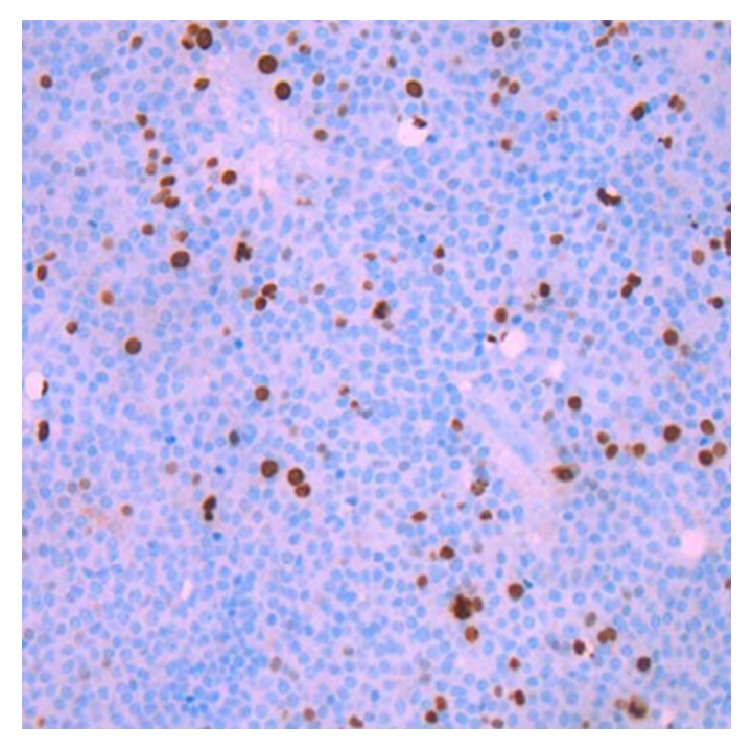
Immunostaining for Ki-67 in pituitary adenoma. Only the dark brown stained nuclei were considered as immunopositive.

Ki-67 LI evaluation was carried out in the Clinic of Pathological Anatomy of the LUHS by the qualified pathologist. The biological markers of proteins were analysed according to the immunohistochemical analysis protocol in paraffin sections by the Ventana BenchMark XT staining procedure (Ventana Medical Systems, Tucson, Arizona, USA). Paraffin sections were dewaxed using Ventana reagent. Ventana Cell Conditioning Solution (pH 8.4) – 100°C 60 min. was used in order to restore antigenic epitopes. Those sections were incubated with monoclonal antibodies for 32 min. at 37°C and identified with Ventana iVIEW DAB Detection Kit. At the end of IHC reaction, sections were contrasted with Gill's Hematoxylin Solution, coloured with bluing reagent of an aqueous solution of buffered lithium carbonate, and covered with glass slides (Figure 2).

*IL-17A*. The serum IL-17A level was measured by enzyme-linked immunosorbent assay (ELISA) according to the manufacturer's instructions (ThermoFisher Scientific Human IL17A ELISA Kit).

Solutions for the analysis were prepared according to the recommendations of the manufacturer. Appropriate solutions of 2000 pg/mL, 1000 pg/mL, 500 pg/mL, 250 pg/mL, 125 pg/mL, 62.5 pg/mL, 31.25 pg/mL, and 0 pg/mL of IL-17A were prepared in order to obtain a calibrated protein standard. 50 *µ*L biotinylated antibody was added to each ELISA 96-well plate. 50 *µ*L of standard or 50 *µ*L of sample was pipetted into the wells twice. The wells were covered and incubated at the room temperature for 2 hours and then washed 3 times with washing buffer solution. 100 *µ*L of streptavidin protein that is conjugated to horseradish peroxidase was added to each well and then plates were covered and incubated for 1 hour. After that, the wells were washed 3 times again with washing buffer solution. Substrate solution was added to each well and they were incubated for 30 min. at the room temperature. The reaction was performed in the dark. Then 100 *µ*L of Stop solution was added to each well and as a result blue colour changed into yellow. Absorption was evaluated by microplate reader at 450 nm and 550 nm wavelengths. The difference between optical densities at these wavelengths was used to assess the concentration. Samples concentration was evaluated by using the standard curve.

*Statistical analysis.* Statistical analysis was performed using the SPSS / W 23.0 software (Statistical Package for the Social Sciences for Windows, Inc., Chicago, Illinois, USA). Normality of the distribution was tested with Shapiro-Wilk and Kolmogorov-Smirnov statistics. Mann-Whitney U test was conducted to compare nonparametric values. The data are presented as absolute numbers with percentages in brackets and as median with min and max values. Differences were considered statistically significant when p<0.05.

## 3. Results

*Ki-67 labelling index. *69 PA tissue samples were investigated. Ki–67 LI was evaluated in 37 women (53.6%) and 32 men (46.4%). The results showed that there was no significant difference in the Ki–67 LI between women and men (p = 0.092).

Immunohistochemistry for Ki-67 revealed a LI < 1% in 49.3% of patients with PA, Ki-67 LI 1% in 24.6%, and Ki-67 LI > 1% in 26.1% of patients. Further analysis showed statistical significance in relation to tumour invasiveness (p = 0.039) (Table 4). However, Ki–67 LI analysis did not reveal any correlation with PA recurrence (p = 0.6) and tumour growth direction (p > 0.05) (Table 5). There were no statistical differences in the Ki-67 LI in relation to the Hardy, modified by Wilson, as well as Knosp classifications (p>0.05) (Table 6).

*The concentration of IL-17A in blood serum*. Serum levels of IL–17A were determined in 60 patients with PA and 64 control subjects. IL-17A analysis showed a significant difference between the median serum IL-17A level in the PA patients group (median 42.12; min 1.95; max 76.80) and the control group (median 8.19; min 0.39; max 74.57). However, analysis did not show any statistical significance comparing different growth types of PA (p > 0.05) (Table 7).

## 4. Discussion

Ki-67 LI is referred to as an indicator of aggressive behaviour in the classification of endocrine tumours [44]. Numerous studies have analysed the impact of Ki-67 LI on PA invasiveness and recurrence [5, 24, 26–35, 45] but only few studies have investigated the associations between the expression of Ki-67 and PA growth direction [27], and data still remain controversial.

In the present study we found that invasive PAs have significantly higher Ki-67 LI compared to noninvasive PAs. Several studies confirmed our results. Thirty-eight invasive and 65 noninvasive PA tumours were analysed in the study conducted by Mastronardi et al. [27]. The research has revealed that Ki-67 LI could be useful marker in the determination of invasive PAs, even if this Ki-67 LI had no significant correlation with tumour size [27]. Wierzbicka-Tutka et al. also have found statistically significant correlation between Ki-67 LI and tumour size and invasiveness [26]. Zaidi et al. have confirmed that Ki-67 LI also could be used as an independent predictor marker for aggressive PAs growth in atypical PAs cases [31]. Knosp et al. [5] have found a significantly higher rate of Ki-67 LI in PAs with surgically observed invasion into the cavernous sinus space compared to noninvasive PAs (p < 0.001). Thapar et al. [28] have analysed Ki-67 LI in 33 invasive and in 37 noninvasive PAs and revealed that invasive PAs had a higher Ki-67 LI than noninvasive PAs. They have established a threshold LI of 3 % to distinguish invasive from noninvasive PAs. Kitz et al. [29] have proven that PAs with histologically proven dural infiltration (real invasiveness) had a significantly higher Ki-67 LI compared to noninvasive PAs (p < 0.001). Another study done by Sadeghipour et al. [30] has analysed Ki-67 LI in 176 patients with PAs, who were treated surgically. Thirty-six patients had recurrent episodes and 11 of 176 patients had an invasive tumour. Scientists revealed that the group of invasive PAs had a higher Ki-67 LI compared to the noninvasive group. However, the difference in Ki67-LI between recurrent and nonrecurring PAs groups was not statistically significant [30].

However, some authors have found no association between the rate of Ki-67 LI and PA invasiveness [33–35]. Yarman et al. [33] concluded that Ki-67 LI expression had no correlation with invasive PAs. Dubois et al. [34] also concluded that Ki-67 LI had no independent prognostic value of PA invasiveness. Gandour-Edwards et al. [35] examined 10 invasive PAs involving the sphenoid sinus and 10 noninvasive PAs. They have not found statistical significant difference between them as well.

Although we have not found any significant correlation between Ki-67 LI and PA recurrence, Paek [45] et al. have demonstrated that PAs with recurrence had a significantly higher Ki-67 LI than adenomas without recurrence (0.56%) (p = 0.027). Landeiro et al. have evaluated 35 patients with giant nonfunctioning PAs. In their survey, 30 patients had Ki-67 LI <3% and, in those patients, recurrence was found in 6.67%. Five patients had Ki-67 LI >3%, and 3 of them were diagnosed with recurrence (60%) [32].

Also we found no correlation between Ki-67 LI and tumour growth direction. Kyung-Il Paek et al. have found that the Hardy classification D (1.08%) had a higher Ki-67 labelling index than the others, but without a statistical significance (*p*=0.460) [45]. We also found that there were no statistical differences in the Ki-67 LI in relation to the Hardy, modified by Wilson, as well as Knosp classifications.

To our knowledge, only one study has explored IL-17A concentration in the serum of patients with PA, but no studies have investigated the association between serum IL-17 and PA growth type. Qiu et al. [42, 43] have investigated IL-17A serum concentration level of 76 PA patients, and they have observed that serum level of IL-17A was significantly higher in patients with invasive PAs than in the group with noninvasive PAs. Serum level of IL-17A was found to be 95.46 ± 34.09 pg/mL in invasive PA patients, 56.26 ± 14.03 pg/mL in noninvasive PA patients, and 23.58 ± 6.55 pg/mL in the control group. In our study we revealed that serum level of IL-17A was significantly higher in patients with PA than in the control group (42.12 versus 8.19 pg/mL, p < 0.001). Unfortunately, the results of our study did not reveal any significant differences between IL-17A serum concentration and PA growth type. We can hypothesize that cytokine IL-17A can be important for PA development but it has no impact on PA growth type.

Overall, the present study demonstrated that impact of Ki-67 LI and IL-17A on PA invasiveness, recurrence, and tumour growth type requires further investigation with increased sample sizes to confirm the association of Ki-67 LI and IL-17A in patients with PA.

## Figures and Tables

**Table 1:** Demographic and clinical data.

**Characteristic**	**PA group** **n=60**	**Control group** **n=64**	**p value**
Gender			

Males, n (%)	22 (36.7)	14 (21.9)	0.070
Females, n (%)	38 (63.3)	50 (78.1)	
Age at onset (years) (min/max/median)	24/83/50	19/82/55	0.465

Symptoms:		NA	-
(1) Visual disturbances (decreased visual acuity, visual field defects, etc.)	39		
(2) Headache	25		
(3) Weakness	9		
(4) Acromegaly (exaggerated growth of the hands and feet, with thick fingers and toes, enlargement of the lower lip and nose, etc.)	8		
(5) Prolactinoma symptoms (disruption of menstrual cycle, milk production unrelated to pregnancy or nursing, etc.)	10		

Recurrence:		NA	-
Absent/present	51/9		

Invasiveness:		NA	-
Invasive/noninvasive	25/35		

Surgery/conservative treatment	55/5	NA	-

**Table 2:** Hardy classification, modified by Wilson [21].

**Grades**	**Sellar floor erosion**	**Extrasellar extensions**				
		Suprasellar extension		Parasellar extension		
		A	B	C	D	E
Grade I	Sella normal or focally expanded; tumor < 10 mm	PA expanding into the suprasellar cistern	Anterior recesses of the third ventricle obliterated	The floor of the third ventricle grossly displaced	An intracranial extension into the anterior, middle, or posterior fossa (intradural)	An intracranial extension into or beneath the cavernous sinus (extradural)
Grade II	Sella enlarged; tumor ≥ 10 mm					
Grade III	Localized sellar perforation					
Grade IV	Diffuse destruction of the sellar floor					

**Table 3:** Knosp classification of pituitary adenoma [5].

Grade 0	No involvement of the cavernous sinus (normal condition)

Grade 1	The tumor pushes into the medial wall of the cavernous sinus, but does not go beyond a hypothetical line extending between the centers of the two segments of the internal carotid artery (noninvasive PA)

Grade 2	The tumor goes beyond hypothetical line, but without passing a line tangent to the lateral margins of the artery itself (noninvasive PA)

Grade 3	The tumor extends laterally to the internal carotid artery within the cavernous sinus (invasive PA)

Grade 4	Total encasement of the intracavernous carotid artery (invasive PA)

**Table 4:** Ki-67 labeling index considering invasiveness of pituitary adenoma.

**PA**	**Ki-67 LI**			**p value** *∗*
	< 1 %	1 %	> 1 %	
Noninvasive PA n=26 (37.7 %)	16 (47.1 %)	2 (11.8 %)	8 (44.4 %)	0.039
Invasive PA n=43 (62.3 %)	18 (52.9 %)	15 (88.2 %)	10 (55.6 %)	

*∗*: *χ*^2^ test; LI: labelling index; PA: pituitary adenoma.

**Table 5:** Ki-67 labelling index in pituitary adenoma tissue considering growth direction of pituitary adenoma.

**PA growth direction**		**Ki-67 LI < 1%** **(n)**	**Ki-67 LI ≥ 1%** **(n)**	**p value** **∗**
Suprasellar growth	Yes	24	26	p = 0.731
	No	10	9	

Paracavernous growth	Yes	10	15	p = 0.245
	No	24	20	

Sphenoidal growth	Yes	16	21	p = 0.281
	No	18	14	

*∗*: Student's t-test; LI: labelling index; PA: pituitary adenoma.

**Table 6:** The Ki-67 LI in relation to the Hardy, modified by Wilson, and Knosp classifications.

**Classification**	**Ki-67 LI, median (IQR)**	**p value** **∗**
Hardy-suprasellar		
0	2.5 (1.00)	0.564
A	2.0 (1.25)	
B	3.0 (2.00)	
C	2.0 (2.00)	

Hardy-parasellar		
0	2.0 (2.00)	0.897
E	3.0 (2.00)	
E,D	2.5 (1.00)	

Knosp		
0	2.0 (2.00)	0.673
1	3.0 (2.00)	
2	2.0 (2.00)	
3	3.0 (1.00)	
4	3.0 (2.00)	

*∗*: Kruskal-Wallis test; LI: labelling index.

**Table 7:** Serum levels of IL-17A in patients with pituitary adenoma and in controls considering growth type of pituitary adenoma.

**PA growth type**	**Group (n)**	**IL-17A concentration (pg/mL)** **min/median/max**	**p value** **∗**
Invasiveness	Invasive (29)	1.95/42.12/76.80	p = 0.701
	Noninvasive (25)	3.10/42.12/76.80	

Recurrence	Recurrent PA (5)	1.95/42.12/76.80	p = 0.664
	Nonrecurrent PA (49)	3.10/42.12/76.80	

Suprasellar growth	Yes (38)	1.95/42.12/76.80	p = 0.234
	No (16)	4.29/39.95/55.12	

Paracavernous growth	Yes (18)	1.95/44.29 /76.80	p = 0.281
	No (36)	3.10/41.04/76.80	

Parasphenoidal growth	Yes (24)	1.95/42.12/76.80	p = 0.700
	No (30)	3.10/42.12/76.80	

**∗**:Mann-Whitney U test; IL-17A: interleukin-17A.

## Data Availability

The data supporting the results reported in the article are not publicly available.
